# Mosquito-Borne Human Viral Diseases: Why *Aedes aegypti*?

**DOI:** 10.4269/ajtmh.17-0866

**Published:** 2018-03-19

**Authors:** Jeffrey R. Powell

**Affiliations:** Yale University, New Haven, Connecticut

## Abstract

Although numerous viruses are transmitted by mosquitoes, four have caused the most human suffering over the centuries and continuing today. These are the viruses causing yellow fever, dengue, chikungunya, and Zika fevers. Africa is clearly the ancestral home of yellow fever, chikungunya, and Zika viruses and likely the dengue virus. Several species of mosquitoes, primarily in the genus *Aedes*, have been transmitting these viruses and their direct ancestors among African primates for millennia allowing for coadaptation among viruses, mosquitoes, and primates. One African primate (humans) and one African *Aedes* mosquito (*Aedes aegypti*) have escaped Africa and spread around the world. Thus it is not surprising that this native African mosquito is the most efficient vector of these native African viruses to this native African primate. This makes it likely that when the next disease-causing virus comes out of Africa, *Ae. aegypti* will be the major vector to humans.

Mosquito-borne viruses (arboviruses) have been afflicting humans for millennia and continue to cause immeasurable suffering. While not the only mosquito-borne viruses, the following four have been the most widespread and notorious in terms of severity of diseases and number of humans affected: the viruses causing yellow fever (YFV), dengue fever (DENV), chikungunya fever (CHIKV), and Zika fever (ZIKV). One enigma concerning these four viruses is that a single mosquito (out of more than 3,500 species), *Aedes aegypti*, has been the vector causing almost all major epidemics outside Africa. Here I discuss why this is the case and how this informs control of these diseases and what it means for stemming future arbovirus pandemics.

## MOSQUITOES

For a mosquito to be a major vector of a human pathogen it must readily use humans as a source of blood meals and live in high densities in close association with humans over a wide geographic area. Many mosquitoes fulfill these criteria. But there is a third criterion: it must allow a human pathogen to grow and infect its saliva. Owing to the limited life span of female mosquitoes, the faster this occurs, the better the vector. For the four arboviruses mentioned, *Ae. aegypti* is the single species outside Africa that best fulfills all these criteria to be an efficient vector.^1–3^ This is likely because of evolutionary history.

*Aedes aegypti* is native to Africa where ancestral populations can still be found breeding in forests and ecotones with larvae in tree holes and adults preferring nonhumans for blood meals.^4,5^ When humans began living in villages and towns in Africa, they started storing water in containers year around, making this an attractive mosquito larval breeding site especially during prolonged dry seasons. *Aedes aegypti* was the African *Aedes* mosquito to exploit this new niche. Females eclosing from containers in villages evolved a preference for blood meals from the most available source: humans. It is this “domesticated” form of *Ae. aegypti* that spread to occupy tropical and subtropical human habitats in six continents today.^6,7^

The timeline for the spread of *Ae. aegypti* is reasonably clear and is consistent with epidemiologic records. Beginning in the sixteenth century, European ships to the New World stopped in West Africa to pick up native Africans for the slave trade^8^ and very likely picked up *Ae. aegypti* and YFV. The first well-documented New World yellow fever epidemics were in Yucatan and Havana in 1648.^9^ Asian populations of *Ae. aegypti* are derived from the New World more recently, on the order of 200 years ago.^7,10,11^ The Mediterranean could well have been an intermediate stop with the introduction to Asia instigated by the opening of the Suez Canal in 1869. The first urban dengue fever in Asia was in the 1890s and chikungunya in 1879.^12,13^

## PRIMATE HOSTS

Humans also first arose in Africa. There is little doubt that man’s closest living relative is the chimpanzee^14^ from which it diverged 6–10 million years ago.^15^ Tatersall^16^ states that “… over the past 2 million years the continent has regularly pumped out new kinds of hominid into other areas of the Old World.” Anatomically modern hominids generally agreed to be *Homo sapiens* appear in Africa 200,000–300,000 years ago.^17,18^ This lineage is thought to have left Africa 60–100,000 years ago and replaced (or hybridized with) other hominids throughout the world.^19^

## THE VIRUSES

Determining the geographic origin of viruses is more difficult than for mosquitoes or primates, nevertheless, there is very good evidence that YFV, CHIKV, and ZIKV are all ancestral to Africa and, although less clear, DENV is likely African in origin.

There are no records of yellow fever existing outside Africa before 1500. Europeans arriving in Africa in the 1300s suffered from yellow fever whereas native Africans often exhibited immunity because of previous exposure.^20^ Molecular analyses of the virus genome confirms an African origin dating back at least 1,500 years.^21^ YFV is known to be transmitted among nonhuman primates by at least 10 native African *Aedes* other than *Ae. aegypti*.^22^

CHIKV was first isolated in 1952 in Tanzania.^23^ A disease recognizable as chikungunya fever is quite clearly described in Asia after 1879,^13^ 10 years after the opening of the Suez Canal hypothesized to have allowed *Ae. aegypti* to enter Asia. Since, CHIKV has spread widely in Asia and more recently in the New World, first detected in 2013.^24^ Outside Africa, *Ae. aegypti* is the major mosquito-vectoring CHIKV, although *Aedes albopictus* can also cause epidemics.^25^ In CHIKV’s native Africa, in addition to *Ae. aegypti*, seven other native African *Aedes* have been implicated as vectoring CHIKV among African primates.^26,27^

ZIKV was first isolated in Uganda in 1947 from a monkey and *Aedes africanus* in the Ziika Forest near Entebbe.^28^ It caused sporadic outbreaks of mild febrile diseases in Africa for the next 60 years. ZIKV was isolated from an *Ae. aegypti* mosquito in Asia in the 1960s.^29^ But it was only in 2007 that hundreds of severe human cases are reported in Asia (Micronesia).^30^ ZIKV’s arrival in Brazil in 2015 set off worldwide concern, if not panic. Zika fever in the New World coincides with the distribution of *Ae. aegypti*. In addition to *Ae. aegypti*, ZIKV has been isolated from seven other native African *Aedes*.^31^

Dengue (DENV) presents a less clear picture of origin. Dengue as a human disease is difficult to unambiguously diagnose before modern medical technologies. A disease with some characteristics of dengue is recorded in the early 1600s in the Caribbean and Panama,^32^ about the same time as the first yellow fever and after *Ae. aegypti* was introduced. Unambiguous dengue is described for the first time almost simultaneously in Africa, Asia, and the Americas in 1779–1788.^12,32^

Unlike the other viruses, DENV was in Asia in the 1700s before *Ae. aegypti* arrived in the late nineteenth century.^33,34^ Thus, a mosquito other than *Ae. aegypti* must have been transmitting DENV in Asia, *Ae. albopictus*, the “Asian tiger mosquito,” being the most likely candidate for human transmission in Asia at that time.^35^ *Aedes albopictus* is a native Asian mosquito that readily bites humans and can live in close association with humans, although generally not as “urbanized” as *Ae. aegypti*.

As to nonhuman circulation of DENV, there is clearly an African primate, sylvan cycle with three African *Aedes* (in addition to *Ae. aegypti*) known to transmit DENV to nonhuman primates.^36^ Evidence for a nonhuman dengue virus cycle in Asia is less clear. Although flavivirus-neutralizing antibodies (attributed to DENV) were detected in arboreal primates in Malaysia, no live virus was isolated from Asian nonhuman primates except from caged monkeys exposed in the canopy of forests in the 1970s and 1980s^37^ after *Ae. aegypti* was in Malaysia. Although a DENV-4 isolate has been made from *Aedes neveus*,^38^ no DENV was found in over 25,000 other arboreal mosquitoes tested in Malaysia.^37^

**Figure 1.**
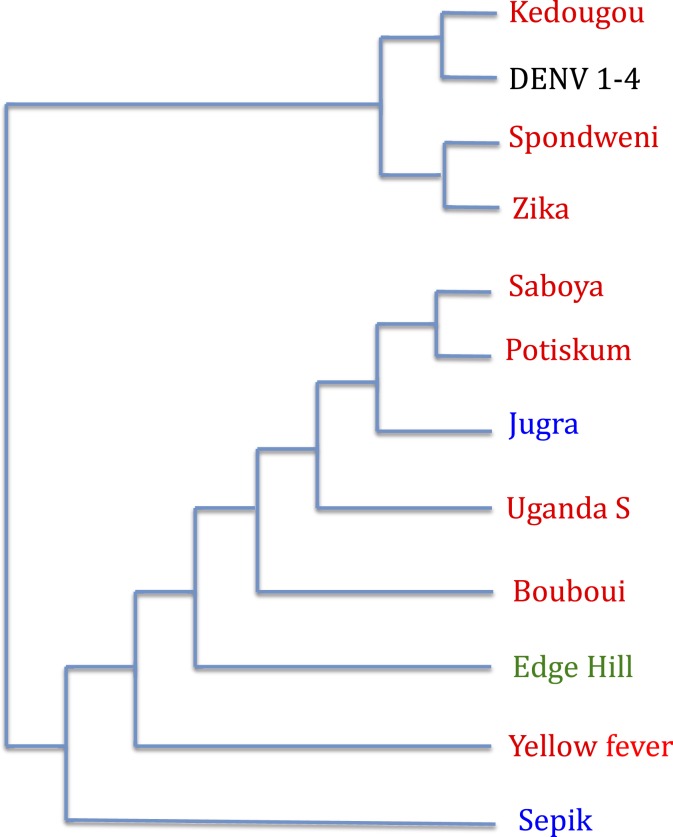
Phylogenetic relationship of flaviviruses transmitted to humans by mosquitoes. Red indicates African origin, blue Asia, and green Australia. “Origin” refers to where the virus was first isolated. Simplified from Gaunt et al.^39^ This figure appears in color at www.ajtmh.org.

Although it is generally thought that the four serotypes of DENV circulating today arose in Asia,^22,36^ this has occurred quite recently. A good case can be made that before this time (> 300 years ago), ancestral DENV was in Africa. Phylogenetic relationships of flaviviruses are a strong argument for this: DENV is in a clade of viruses most of which are known to be African (Figure 1).^39^ The branch leading to present day serotypes of DENV was an African lineage closely related to ZIKV. So even if the diverse serotypes of DENV were not all African in origin, it is very likely that further back in time, DENV’s predecessors were in Africa. So like the other three viruses, this implies DENV (or its immediate ancestors) had a long history of transmission by African mosquitoes to African primates.

### Implications for public health.

Two important conclusions can be drawn from these observations.

First, given that a single mosquito is the primary vector of multiple human diseases, rather than fighting each disease individually as it comes to the fore, control the single mosquito species. This begs the question of just what it means to “control” a mosquito. It is not the purpose here to review or suggest mosquito control methods, rather to point out that multiple diseases can be reduced or eliminated by targeting a single mosquito.

Second, when the next Zika-like epidemic hits, it is likely to be caused by a virus now present in Africa and will be transmitted to humans primarily by *Ae. aegypti*. History predicts this. Are there more such viruses in Africa? Owing to the efforts of the Rockefeller Foundation, collections of more than 4,000 mosquito virus isolates were amassed, the majority from Africa.^40^ The four discussed here are only the tip of the iceberg of African mosquito–borne viruses. And these are the known mosquito-borne viruses. One can only speculate on how many mosquito-borne viruses lurk in Africa and how many cause diseases in African primates like man. So by controlling *Ae. aegypti*, not only would the major mosquito-borne viral diseases presently afflicting humans be controlled, but also future emergence of new viruses from Africa would be stopped or at least inhibited.
